# Application of Weibull model for survival of patients with gastric cancer

**DOI:** 10.1186/1471-230X-11-1

**Published:** 2011-01-07

**Authors:** Hui P Zhu, Xin Xia, Chuan H Yu, Ahmed Adnan, Shun F Liu, Yu K Du

**Affiliations:** 1 Department of Maternal and Child Health, School of Public Health, Tongji Medical College, Huazhong University of Science and Technology, Wuhan, PR China; 2 Department of Epidemiology and Statistics, School of Public Health, Wuhan University, Wuhan, PR China; 3 Department of General Surgery, Tongji Hospital, Tongji Medical College, Huazhong University of Science and Technology, Wuhan, PR China

## Abstract

**Background:**

Researchers in the medical sciences prefer employing Cox model for survival analysis. In some cases, however, parametric methods can provide more accurate estimates. In this study, we used Weibull model to analyze the prognostic factors in patients with gastric cancer and compared with Cox.

**Methods:**

We retrospectively studied 1715 patients with gastric cancer. Age at diagnosis, gender, family history, past medical history, tumor location, tumor size, eradicative degree of surgery, depth of tumor invasion, combined evisceration, pathologic stage, histologic grade and lymph node status were chosen as potential prognostic factors. Weibull and Cox model were performed with hazard rate and Akaike Information Criterion (AIC) to compare the efficiency of models.

**Results:**

The results from both Weibull and Cox indicated that patients with the past history of having gastric cancer had the risk of death increased significantly followed by poorly differentiated or moderately differentiated in histologic grade. Eradicative degree of surgery, pathologic stage, depth of tumor invasion and tumor location were also identified as independent prognostic factors found significant. Age was significant only in Weibull model.

**Conclusion:**

From the results of multivariate analysis, the data strongly supported the Weibull can elicit more precise results as an alternative to Cox based on AIC.

## Background

Gastric carcinoma (GC) is one of the leading causes of cancer-related death in the world [1-3], and it is the most common malignant tumor in Asia, Eastern Europe, and South America [4,5]. In Japan, stomach cancer ranks the first place in women and the second place in men with respect to the cause of death from malignant tumor [6,7]. Although age-standardized mortality rate of GC has decreased in China now, it is still the third most common causes of death in men after lung and liver cancer, and the fifth in women. For locally advanced gastric cancer patients, the 5-year survival rate is below 20% and it is about 30% for those undergoing surgical treatment. Even after taking curative resections, only 30-50% of the patients can survive after 5 years [8,9]. In terms of treatment for gastric cancer, surgery is considered as the best way for achieving good outcomes [10]. However, there are still lots of challenges for gastrointestinal doctors to conquer. It is estimated that at least 80% of patients recur disease even after they took curative gastric resections. Previous studies tried to find out clinic-pathological factors and socio-demographic characteristics associated with high recurrence rate. These studies had drawn conflicting results with respect to independent prognostic factors affecting survival of patients with GC [1].

Parametric methods which include the exponential, Weibull, lognormal, gamma and extreme value distributions have been widely used in fitting survival data [11]. Cox semi-parametric method [12] has also been extensively used for modeling such data. These methods are presented to account for the relationship between survival and some concomitant variables such as age, gender, family history of gastric cancer, or diagnostic characteristics. Actually, in the medical sciences, researchers lean to use Cox semi-parametric method instead of parametric methods to analyze survival data. For example, there were studies that have been done to assess the effect of clinic-pathological and demographic factors on survival of patients with stomach cancer using Cox model to find pertinent relationship between survival time and the variables [13-15]. The primary reason is that there seem to be fewer assumptions in the use of Cox semi-parametric method. In some circumstances, however, parametric methods can provide more accurate estimates [16,17]. Many of the parametric models such as Weibull are accelerated failure time models. Weibull allows more flexibility than the Cox semi-parametric model because the associated hazard rate is not constant with respect to time. Also, we use maximum likelihood process to estimate the unknown parameters and its interpretation and technique are familiar for researchers.

In this study, we aimed to evaluate the potential prognostic factors that may affect the survival of patients with gastric cancer employing Weibull model, and to compare analytic results with Cox's proportional hazard model.

## Methods

### Data Sources

We reviewed hospital database of 1,814 patients with gastric cancer who underwent surgical treatment in Tongji hospital in Wuhan, China, during the years 1995 to 2006. We retrospectively reviewed their medical records and excluded 99 patients for incomplete medical document. Finally, 1,715 patients were enrolled in our study. Thereafter, all the patients were observed through a programmed followed-up schedule. Survival information was collected through telephone interviews with patients and/or their relatives who were at home at the time of interview. This study was approved by the Ethics Committee of Huazhong University of Science & Technology.

Gastric cancer stage was evaluated according to the International Union Against Cancer (UICC) TNM classification of malignant tumors [18]. Survival analysis was based on the clinical and pathologic variables, which were sub-layered into family history of GC, histologic grade (well, moderately and poorly differentiation), tumor location (upper, middle and lower) in the stomach, the stage of the carcinoma (I, II, III, IV), depth of tumor penetration (T1, T2, T3, and T4) as defined by the American Joint Committee on Cancer (AJCC), N categories on the basis of the number of metastatic lymph nodes (pN0: 0, pN1: 1-6, pN2: 7-15, pN3: > 15) defined by the International Union Against Cancer (UICC) and the American Joint Committee on Cancer (AJCC) in the 5th edition of the TNM system in 1997 [19].

### Statistical Analysis

Statistic calculations were performed using statistical software SAS, version 9.1. Quantitative result was expressed as the mean ± standard deviation (SD). Univariate analysis was conducted using the Kruskal-wallis and *t *test. Differences at *P *< 0.05 were considered significant. Covariates that were identified as significant factors throughout the univariate analysis were selected for multivariate analysis, which was performed employing Weibull and Cox's proportional hazard model to build the prognostic indicators of survival in patients with gastric cancer. A plot of the log of the negative log of the estimated survivor function against log time (by specifying LLS) was drawn. LLS plot can provide a visual check of the appropriateness of the Weibull model for the survival data [20]. HR (hazard rate) and the AIC (Akaike Information Criterion) were used to compare the efficiency of models between Weibull and Cox model. The AIC is a measure of the goodness of fit of the model estimated that proposed by Akaike in 1974 [21] and is a practical way of trading off the complexity of an estimated model against how well the model fits the data. Lower AIC indicates better likelihood.

## Results

### Clinical and pathologic features

Patient characteristics were detailed in Table 1. A total number of 1715 patients with gastric cancer entered to this study, 465(27.1%) were women and 1250(72.9%) man. The mean age at diagnosis was 57.5 ± 10.9 years (range = 21~90 years). Evidence of family history and past history of GC were seen in 284 patients (16.6%) and 457 patients (26.6%) respectively. Of the total patients, 1315 patients (78.8%) had tumour size ≥40 mm, 492 patients (28.7%) diagnosed with stage IV of gastric cancer. Tumors were located in the lower third stomach in 1086 patients (63.3%), in the middle third of the stomach in 281 patients (16.4%), in the upper third stomach in 193 patients (11.3%), and whole stomach 155 patients (9.0%). Among all the patients, 809 (47.8%) patients received utterly eradicative degree of surgery. Tumors were classified as well differentiated in 521 patients (30.3%), moderately differentiated in 253 patients (14.8%), and poorly differentiated in 941 patients (54.9%). Lymph node involvement defined by AJCC classifications included 629 patients with N0 category, 717 patients with N1 category, 272 patients with N2 category, and 97 patients with N3 category. AJCC T1 about depth of invasion was identified in 145 patients (8.5%), AJCC T2 in 879 patients (51.3%), AJCC T3 in 549 patients (32.0%), and AJCC T4 in 142 patients (8.3%).

**Table 1:** Clinic-pathological characteristics of the patients with gastric cancer

Factors	Categories	No. of patients (%)	*P *value
Gender	Female	465(27.1)	0.301
	Male	1250(72.9)	
age		57.5 ± 10.9	<0.001
Past medical history	No	1258(73.4)	0.022
	Yes	457(26.6)	
Family history of gastric cancer	No	1431(83.4)	0.431
	Yes	284(16.6)	
Location of tumor	Lower third	1086(63.3)	<0.001
	Middle third	281(16.4)	
	Upper third	193(11.3)	
	Whole stomach	155(9.03)	
Eradicative degree of surgery	Utterly	809(47.8)	<0.001
	Relatively	473(27.6)	
	Palliative	433(25.2)	
Tumor size(mm)	<40	364(21.2)	<0.001
	≥40	1315(78.8)	
Stage	I	301(17.5)	<0.001
	II	425(24.8)	
	III	497(29.0)	
	IV	492(28.7)	
Combined evisceration	No	1323(77.1)	<0.001
	Yes	392(22.9)	
Histologic grade	Well differentiated	521(30.3)	<0.001
	Moderately differentiated	253(14.8)	
	Poorly differentiated	941(54.9)	
Depth of invasion	T1	145(8.5)	<0.001
	T2	879(51.3)	
	T3	549(32.0)	
	T4	142(8.3)	
Lymph node status	N0	629(36.7)	<0.001
	N1	717(41.8)	
	N2	272(15.9)	
	N3	97(5.7)	

T1, Tumour invades lamina propria or submucosa;

T2,Tumour invades muscularis propria or subserosa;

T3: Tumour penetrate serosa without invasion of adjacent structures;

T4: Tomour invades adjacent structures;

N0, Metastasis in 0 regional lymph nodes;

N1, Metastasis in 1 to 6 regional lymph nodes;

N2, Metastasis in 7 to 15 regional lymph nodes;

N3, metastasis in more than 15 regional lymph nodes.

### Distribution of the survival time

**Figure 1:**
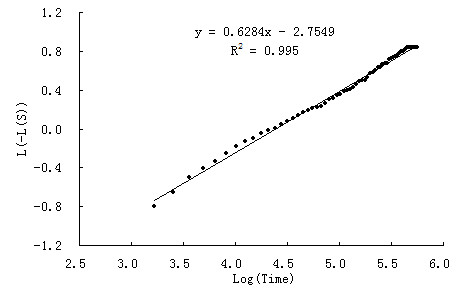
**Log of negative Log survivor function estimates**.

Usually, a first step in the analysis of survival data is the estimation of the distribution of the survival time. Figure 1 displays the graph of the log (-log (estimated survival function)) against log (failure time), i.e. LLS plot. If the Weibull model is appropriate, the LLS curve should be a straight line that does not necessarily go through the origin. This is because S(t) = exp(-(la t)^alpha) holds if -log S(t) = (la t)^alpha, or if log(-log S(t)) = alpha log(la) + alpha log t. The slope of the line in the LLS plot is the Weibull shape parameter alpha and the intercept is alpha log (la). In this study, the lls plot looks approximately linear which suggests graphically that the survival-time distribution considered is Weibull. Moreover, the value of intercept and scale were -3.324 and 1.362, respectively, and alpha value is 0.734 given from SAS results.

### Multivariate Weibull and Cox Analysis of Prognostic Factors

In univariate analysis, age(*P *< 0.001), past medical history(*P *= 0.022), tumor size (*P *< 0.001), histologic grade (*P *< 0.001), tumor location(*P *< 0.001), eradicative degree of surgery(*P *< 0.001), tumor stage (*P *< 0.001), combined evisceration (*P *< 0.001), depth of invasion (*P *< 0.001), and lymph node status (*P *< 0.001) were found significant factors that have influence on overall survival in all gastric cancer patients who underwent surgical treatment (Table 1). Variables shown to be of statistical significance in univariate survival analysis were further assessed by Weibull and Cox multivariate analysis. According to the results from both Cox and Weibull model patients with the past history of having gastric cancer had the risk of death increased significantly in term of hazard ratio in Cox regression and Weibull model followed by poorly differentiated and moderately differentiated in histologic grade (*P *< 0.05). Eradicative degree of surgery, pathologic stage, depth of tumor invasion and location of tumor were also identified as independent prognostic factors found significant. Age is significant in Weibull model but insignificant in Cox model for multivariate analysis (Table 2, 3). Neither Cox nor Weibull model in both univariate and multivariate analysis show any evidence about significant differences in gender and family history of cancer. In multivariate models, the Weibull model had the best fit with respect to lower AIC (Table 3).

**Table 2:** Multivariate analysis of Weibull parametric model with prognostic factors

*Characteristics*	*β*	*χ^2 ^value*	*P *value
Intercept	0.76	0.53	0.467
Age	-0.03	6.27	0.012
Past medical history	-0.11	7.13	0.008
Location of tumor	-	25.40	<0.001
Lower third	0.40	8.83	0.003
Middle third	0.41	7.34	0.007
Upper third	-0.17	1.08	0.299
Whole stomach*	0	-	-
eradicative degree of surgery	-	20.62	<0.001
Utterly	1.00	83.46	<0.001
Relatively	0.91	42.27	<0.001
Palliative*	0	-	-
Histologic grade	-	12.51	0.002
Well differentiated	-0.08	0.80	<0.001
Moderately differentiated	0.34	9.30	0.082
Poorly differentiated*	0	-	-
Depth of invasion	-	49.11	<0.001
T1	0.77	10.55	0.001
T2	0.22	2.48	0.115
T3	0.21	2.29	0.130
T4*	0	-	-
Stage	-	22.41	<0.001
I	0.62	8.27	0.004
II	0.76	21.22	<0.001
III	0.27	6.23	0.013
IV*	0	-	-

*stands for a control group, and the rest compare with the control

**Table 3:** Multivariate analysis of Cox and Weibull model with prognostic factors

*Characteristics*	Cox(AIC = 4534.21)	Weilbull(AIC = 1693.28)
	HR (CI: 95%)	HR (CI: 95%)
Age	1.01 (0.98-1.03)	1.03* (1.01-1.06)
Past medical history		
No	1	1
Yes	1.17* (1.03-1.33)	1.22* (1.05-1.40)
Location of tumor		
Lower third	1	1
Middle third	0.93 (0.74-1.18)	0.99 (0.86-1.25)
Upper third	1.47* (1.12-1.93)	1.35* (1.19-1.53)
Whole stomach	1.45* (1.08-1.93)	1.47* (1.21-1.75)
eradicative degree of surgery		
Utterly	1	1
Relatively	1.03 (0.77-1.39)	1.79*(1.64-1.92)
Palliative	2.16*(1.71-2.73)	4.07*(3.85-4.34)
Histologic grade		
Well differentiated	1	1
Moderately differentiated	1.12*(1.05-1.19)	1.14*(1.08-1.24)
Poorly differentiated	1.25*(1.18-1.33)	1.34*(1.17-1.55)
Depth of invasion		
T1	1	1
T2	1.97*(1.53-2.54)	2.40*(2.10-2.53)
T3	2.19* (1.68-2.86)	2.77* (2.53-2.96)
T4	2.50*(1.82-3.44)	3.15*(3.20-3.99)
Stage		
I	1	1
II	0.97 (0.57-1.63)	1.15 (0.91-1.42)
III	1.57 (0.97-2.56)	1.93* (1.66-2.25)
IV	2.06* (1.21-3.51)	3.03* (2.76-3.80)

*significant at the 5% level

HR, hazard ratio; CI, confidence interval

AIC, akaike information criterion

## Discussion

In the field of medical sciences, researchers are interested in estimating the survival model with the vector of explanatory variables using Cox proportional hazard model more than parametric models. When conducting survival analysis employing Cox model, it is necessary to check the underlying assumptions. Cox model assumes that changes in levels of the independent variables will produce proportionate changes in the hazard function, independent of time. Also, it assumes a log-linear relationship between the hazard function and the time and any number of metric and/or nonmetric variables. In fact, however, assumptions that Cox proportional hazards modeling required may not be plausible in many situations [22], especially in biomedical field. If these assumptions do not hold, the Cox model will lead to unreliable conclusions. Unfortunately, according to Altman's review of survival analyses in cancer journals, only 5 percent of all studies using the Cox model check the underlying assumptions [23]. In the meantime, various parametric models such as Weibull and Lognormal had been developed to analyze survival data. These models can provide the interpretation based on specific distributions for survival time without need the proportional hazard assumptions. If survival times are Weibull or exponentially distributed, the analysis using parametric methods is more powerful [16]. This means under certain circumstances, parametric models like Weibull, Exponential and Lognormal can elicit more accurate results than Cox model. Since population survival times are usually exponentially or Weibull distributed in the field of medicine, therefore, a parametric model will be more efficient and easier to specify than the corresponding semiparametric or nonparametric one and are more flexible as it allows easy incorporation of covariates. Several studies applying parametric models to evaluate prognostic factors affecting survival time of patients with cancer prove that parametric models offer advantages over Cox model [16,24].

The purpose of this study was to explore the comparative performance of Weibull model and Cox model in a survival analysis of patients with gastric cancer. We used Akaike Information Criterion (AIC) to evaluate the two models. In a recent review of survival analyses, it was found that many studies have indicated clinical and pathologic characteristics of patients as explanatory variables with respect to survival [25-27]. In this study, we investigate the effects of age at diagnosis, gender, family history of cancer, past medical history, location of tumor, tumor size, eradicative degree of surgery, depth of tumor invasion, pathologic stage, histologic grade and lymph node status on survival time. Both Weibull and Cox multivariate analysis showed that with the past history of having gastric cancer, patients had significantly increased risk of death followed by the poorly differentiated and moderately differentiated in histologic grade. In addition, eradicative degree of surgery, pathologic stage, depth of tumor invasion and location of tumor were identified as independent prognostic factors of patients with GC as well. In our results, gender showed no impact on survival rate. But, some studies found that better survival rate for women [28], another reported that the consistently lower survival for stomach cancer among women [6].

Age at diagnosis was a strong and independent covariate for survival of patients with GC, and young patients had better survival as indicated by previous report [29]. Tumor size is a significant factor that had impact on the survival probability of patients in univariate analysis, which is similar to some other studies [30,31]. Depth of invasion was another outstanding prognostic indicator in both univariate and multivariate analysis. Our finding is in conformity with previous reports showed that depth of invasion has an influence on patient's survival [32,33]. Stage at diagnosis was strongly associated with prognosis in our study, which is a finding repeated in several other studies [34-36]. Previous reports have demonstrated that the number of metastatic lymph nodes was a powerful predictor of survival. Patients with metastases to 7 or more lymph nodes (N2, N3) had a notably worse outcome as opposed to patients with no lymph node metastases or metastases in 1 to 6 nodes [1,2,37]. However, our findings are not consistent with those previously reported showed by multivariate analysis. Furthermore, our study results suggested that histological classification was an independent predictor of survival.

In our study, age is significant in Weibull model, but it is insignificant in Cox regression for multivariate analysis. Cox model will only be used when the hazard rate is constant with respect to time, but from the Figure 1 in our study we can see that the survival-time distribution was Weibull distribution, so it is more accurate to use Weibull model. The evaluation criteria also indicated Weibull model to be more efficient in comparison to Cox in multivariate analysis. The findings strongly showed Weibull was the perfect model and might lead to more precise results.

## Conclusions

Our study showed that age at diagnosis, past medical history, stage, eradicative degree of surgery, histologic grade, depth of tumor invasion and location of tumor were prognostic factors for survival in patients with GC. It can be concluded that the early detection of patients at younger age and in primary stages and histologic grade may have positive effect on patients with stomach cancer and be important to decrease the survival time. Also, from the results of multivariate analysis, the data strongly supported the Weibull model can elicit more precise results as an alternative to Cox.

## Competing interests

The authors declare that they have no competing interests.

## Authors' contributions

HPZ and XX participated in the design of the study and data collection and helped to draft the manuscript. CHY performed the statistical analysis. AA and SFL participated in data collection and drafted the manuscript. YKD conceived of the study, and participated in its design and coordination. All authors read and approved the final manuscript.

## Pre-publication history

The pre-publication history for this paper can be accessed here:

http://www.biomedcentral.com/1471-230X/11/1/prepub
